# Effects of Years of Operation of Photovoltaic Panels on the Composition and Diversity of Soil Bacterial Communities in Rocky Desertification Areas

**DOI:** 10.3390/microorganisms13061414

**Published:** 2025-06-17

**Authors:** Wenjiao Gao, Yungen Liu, Jun Hu, Zhifeng Luo, Jiaxu Zhang, Yan Wang

**Affiliations:** 1 School of Soil and Water Conservation, Southwest Forestry University, Kunming 650224, China; gaowenjiao288@163.com (W.G.);; 2 Key Laboratory of Ecological Environment Evolution and Pollution Control in Mountainous & Rural Areas of Yunnan Province, Kunming 650051, China; 3 Zhanyi Karst Ecosystem Observation and Research Station, Qujing 650224, China

**Keywords:** photovoltaic operation, rocky desertification, soil bacterial community, microbial diversity, soil enzyme activity

## Abstract

Soil bacterial community composition and diversity can be an important bioindicator for assessing ecosystem stability, and photovoltaic (PV) shading is a key factor influencing soil bacterial communities in rocky desertification areas; however, how the composition and diversity of soil bacterial communities change with PV operation duration remains unclear. Focusing on the experimental demonstration site of Shilin ecological photovoltaic (PV) power plant in Yunnan Province, we compared soil properties under PV arrays and non-PV control areas with different operation durations (7 and 13 years). The results showed that long-term PV operation significantly increased soil TN and TK content compared to CK, while increasing Ure and ALP activities, but inhibiting CAT activity and decreasing soil moisture, pH, SOC, and TP. High-throughput sequencing revealed stable dominant bacterial phyla (e.g., *Aspergillus*, *Acidobacteriota*) and beneficial genera (e.g., *RB41*, *Sphingomonas*), with an increase in relative abundance of *Bacillota-like* phyla but a decrease in *Acidobacterium*. The α-diversity (ACE, Chao1 index) and β-diversity of soil bacteria greatly increased with years of PV operation, reaching a maximum in the 13-year PV operation area. Correlation analyses showed that differences in soil bacterial communities in regions with different years of PV operation were mainly influenced by differences in PH and enzyme activities.

## 1. Introduction

As a typical representative of third-generation renewable energy technology, the large-scale application of photovoltaic energy system has become the core driving force of global energy transformation [1]. Especially in rocky desertification areas, which are rich in light and heat resources, the total solar radiation reaches 4500–5800 MJ/m^2^, and they are a strategically preferred area for the construction of centralized photovoltaic (PV) power plants. However, the cascading risk of “shade effect—nutrient restructuring—soil microbiome disturbance” triggered by the construction of large-scale PV arrays, as well as the potential impacts of the PV panels’ operating life on its natural environment, are uncertain, so the contradiction between coordinating PV development and ecosystem protection needs to be solved. It was found that the construction and operation of PV panels affects vegetation, soil physical and chemical properties, and soil microbial communities by changing wind direction, wind speed, and precipitation pattern [2]. As the core function carriers of soil ecosystems, soil bacterial communities can play an invaluable role in the regulation of carbon flu [3], humification of apoplastic materials, maintenance of soil structure formation, regulation of biodiversity, and maintenance of ecosystem stability [4]. Therefore, the study of the influence of regional soil environmental factors and enzyme activities on soil bacterial communities and diversity under PV panels of different ages can not only enrich the response to the impact of PV power stations on the soil environment, but also help to comprehensively play a good role in the ecological restoration of soil microorganisms.

At present, domestic and foreign research on the ecological effects of PV power plant construction is mainly concentrated in the geographical distribution of the Northwest Arid Zone and Inner Mongolia grassland ecosystem, and most of the research content is aimed at the microclimate changes triggered by the shading of photovoltaic panel [5,6]. However, in high ecological vulnerability areas such as karst desertification and coastal salt marshes, the long-term effects of PV operation years on soil physicochemical properties, enzyme activity, bacterial community stability, and other biological processes have rarely been studied [7]. In a study of arid sandy ecosystems, Adler et al. found that the construction of photovoltaic power plants can significantly affect soil moisture and nutrient status by altering the microhabitat [8], while the shading effect of the photovoltaic panels can reduce the surface moisture and nutrient [9]. In addition, the shading effect of PV panels can reduce the evaporation of water from the surface, which will have a direct or indirect impact on the soil material cycle and microbial community [10]. In addition, some studies have found that the construction of photovoltaic power plants can not only significantly increase the diversity of soil bacteria and optimize the bacterial community structure, but also increase the relative abundance of dominant bacterial phyla under photovoltaic panels [11]. In contrast, some studies have shown that the construction of PV power plants does not have a significant effect on soil microbial α-diversity, which may be due to the fact that the composition and diversity of soil bacterial communities are influenced by many biotic and abiotic factors [12]. Although many studies have been conducted to extensively discuss the effects of changes in soil environmental factors on soil bacterial communities under PV panel areas and areas without PV panels, there is still a relatively limited amount of research on the differences in soil physicochemical properties and enzyme activities caused by differences in the number of years of operation of PV panels. In most PV sites, the construction time of PV arrays can be inconsistent when planning PV parks, due to a combination of factors such as terrain environment and local productivity [13]. It is found that the different operating years of PV panels reorganize soil nutrients in the area, and this reorganization directly or indirectly affects the soil enzyme activity and the microenvironment under the panels, thus forming differences [14]. Therefore, it is necessary to carry out further research on the scientific issue of the influence of soil physicochemical properties and enzyme activities on soil bacteria with different operating years of PV panels in rocky desertification areas.

In this study, we analyzed the characteristics and interactive relationships of soil environmental factors, enzyme activities, and soil bacterial communities under different operating years of PV panels in the experimental demonstration base of Yunnan Shilin Ecological Photovoltaic Power Station in Yunnan Province, with the aim of providing a theoretical paradigm and practical anchors for the synergistic development of photovoltaic construction and ecological safety.

## 2. Methods and Materials

### 2.1. Overview of the Study Area

**Figure 1:**
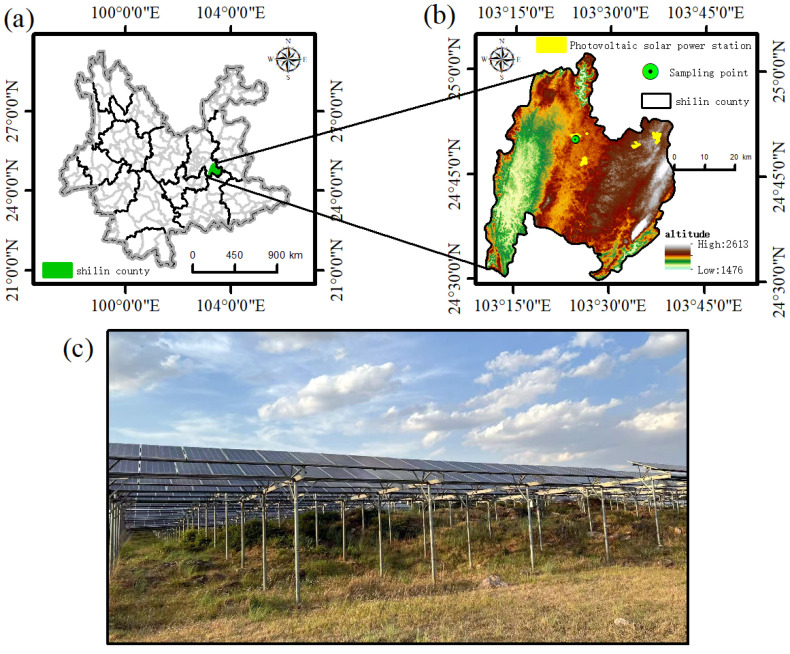
Location of study area and sample geomorphology. (**a**) Kunming City, Yunnan Province. (**b**) Shilin County, Kunming City, Yunnan Province. (**c**) Photovoltaic solar energy base sampling site.

The study area is located in Shilin Yi Autonomous County, Kunming City, Yunnan Province, Shilin County belongs to belongs to the northern subtropical climate zone (Figure 1). The average temperature is 15.9 °C and the frost-free period is more than 250 days There are four seasons, with dry and wet seasons having distinct climatic characteristics. The average annual precipitation is 924.2 mm, the annual evaporation of 1909.4 mm, the seasonal distribution of precipitation is not uniform, and the relative humidity is 70%. This area is the experimental demonstration base of Yunnan Shilin Ecological PV Power Station (Table 1), with a geographical location of 103°24′ E, 24°50′ N, an altitude of 1900 m, scarce clouds, long sunshine time, average annual sunshine time of 2193.3 h, abundant light resources, and a total annual average solar radiation of 5000~6000 MJ·m^−2^. The soil type is mainly dominated by red loam and karstic calcareous soil, and the sample plot vegetation mainly consists of ryegrass (*Lolium perenne* L.), crested fern (*Pteris cretica* L. var. nervosa), and white fescue (*Imperata cylindrica*). The first phase of the project was put into operation in 2010, with a total installed capacity of 10 MW, and the second phase of the project was put into operation in 2016, with a total installed capacity of 56 MW. The project mainly adopts crystalline silicon PV modules for grid-connected power generation, with a column spacing of more than 4 m and a row spacing of more than 6 m. The height of the front gable of the PV panels from the ground is 1.3 m, the height of the rear gable from the ground is 1.9 m, and the tilt angle is 24°. The base site covers an area of more than 2000 mu, which is a typical rocky desertification land in the southwest region.

### 2.2. Sample Collection and Processing

At the end of May 2024, the area under the PV panels with 2 different operating years and the control area without erected PV panels were selected for this study. Three replicate sample plots of 25 × 25 m were set up in each area, with a 15 m separation between sample plots, for a total of 9 sample plots. Five 1 × 1 m sample plots were randomly selected directly below the PV panels in each sample plot for vegetation survey and soil sampling. The serpentine sampling method was selected, using a small shovel to collect five soil samples from 0 to 20 cm depth within each sample plot, which were mixed to make one sample, totaling 45 samples. Ring blade soil and aluminum box soil were also collected and quickly brought back indoors to determine soil moisture content (%) and record information on habitat factors at each sample site, such as elevation and soil type. Each sample was processed as follows: one sample of about 15 g of soil was kept in a sterile bag under refrigeration and handed over to Shanghai Lingen Biotechnology Co., Ltd. (Shanghai, China) for testing; the remaining samples were left indoors to dry naturally, then gravel, plant roots, and debris were removed for the determination of physical and chemical properties of the soil.

### 2.3. Determination of Soil Physical and Chemical Factors and Enzyme Activities

Soil physicochemical property indexes were determined with reference to <Soil Agrochemical Analysis> [15]. Moisture content was determined by drying method; SOC content was determined by potassium dichromate volumetric method in LY/T 1237-1999 <Determination of Organic Matter in Forest Soils> [16]; TN content was determined by Kjeltec method (fully automated KjeltecTM 8400, FOSS, Denmark) in LY/T 1228-2015 <Determination of total nitrogen in forest soil>; TP content was determined by alkali fusion-molybdenum antimony colorimetric method in LY/T 1232-2015 <Determination of total phosphorus in forest soil>; TK content was determined by alkali fusion-flame photometry; soil pH was determined by potentiometric method in LY/T 1239-1999 <Determination of pH in Forest Soil> (LeiMagnet pHS-25 pH meter, Shanghai Yidian Scientific Instrument Co., Ltd., Shanghai, China), with a water–soil ratio of 2.5:1. Soil enzyme activities were determined with reference to Guan [17], in which ALP activity was determined by the nitrobenzene substrate colorimetric method for ALP activity; indophenol blue colorimetric method for Ure activity [18]; and potassium permanganate titration method for CAT activity.

### 2.4. High-Throughput Sequencing of Soil Bacteria

The total DNA of soil samples was extracted using the MOBIO PowerSoil DNA Isolation Kit following the manufacturer’s instructions (MOBIO Laboratories, Carlsbad, CA, USA). The purity of the extracted bacterial DNA was assessed via 2% agarose gel electrophoresis [19], and the DNA concentration was measured using a NanoDrop 2000 (Thermo Fisher Scientific, Wilmington, DE, USA). The DNA was then amplified via PCR using the primers 806R (5′-GGACTACHVGGGTWTCTAAT-3′) and 338F (5′-ACTCCTACGGGAGGCAGCA-3′). The PCR products were further purified to construct sequencing libraries, which were sequenced on the Illumina MiSeq platform (Major Bio, Shanghai, China). The sequencing results were processed and stored using FLASH software (FLASH v1.2.11), and operational taxonomic units (OTUs) were clustered at a 97% similarity threshold for subsequent statistical analysis [20].

### 2.5. Data Analysis

In this study, Excel 2022 was used for the preliminary organization of the data, and one-way ANOVA (one-way analysis of variance) was performed on the environmental factors and enzyme activities by IBM SPSS Statistics 20, and descriptive statistics, analysis of variance (ANOVA), and Pearson’s correlation analysis (Pearson’s correlation analysis) were used for the data processing. The alpha diversity of soil microorganisms and plants was characterized using the Shannon index and species richness, and bacterial community differences were examined by principal coordinate analysis (PCA). Bacterial phylum level differences were analyzed by linear discriminant analysis (LDA) using LEfSe analysis (linear discriminant analysis effect size) of the Micromeritics biotechnology cloud platform in order to determine the species whose relative abundance had significant intergroup difference [21]. In order to analyze the spatial and temporal dynamics of the soil environmental factor–enzyme activity–microbiome interaction network, this study integrated the Mantel test and multivariate statistical methods to quantify the multidimensional driving effects of soil physicochemical parameters and biochemical activities on the bacterial community structure (β-diversity) and the diversity succession pattern across the gradient of years of operation of the photovoltaic facility.

## 3. Results

### 3.1. Effect of Years of Operation of Photovoltaic Panels on the Physicochemical Properties of Soil

**Figure 2:**
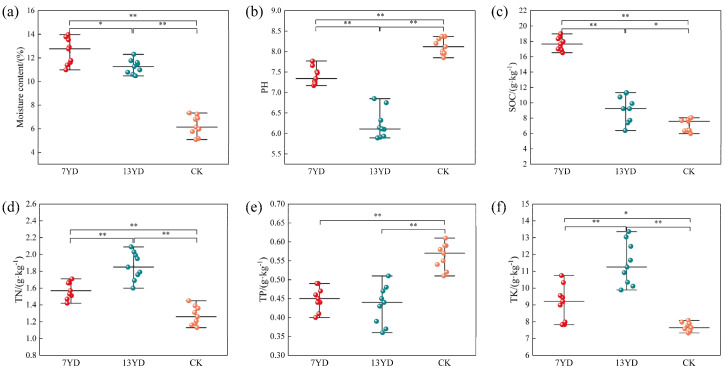
Temporal variation of soil environmental factors along PV array operational duration (* *p* < 0.05, ** *p* < 0.01). (**a**) SWC: gravimetric soil water content. (**b**) pH: soil pH. (**c**) SOC: soil organic carbon. (**d**) TN: total nitrogen. (**e**) TP: total phosphorus. (**f**) TK: total potassium.

As shown in Figure 2, the soil physicochemical factors in the study area exhibited differential responses to photovoltaic (PV) panel shading across operational durations. Temporal analysis (Figure 2a–f) demonstrated that prolonged PV operation significantly decreased SWC, pH, SOC, and TK contents (*p* < 0.05) while increasing total nitrogen (*p* < 0.05). Compared to unshaded areas, PV-shaded zones showed significantly higher SWC and SOC (*p* < 0.01), with 35% and 33% increases in TN and TK, respectively, but 17% and 21% reductions in pH and total phosphorus (*p* < 0.05), indicating that PV shading drives soil nutrient redistribution through modified hydrothermal balance.

### 3.2. Effect of Years of Operation of Photovoltaic Panels on Soil Enzyme Activity

**Figure 3:**
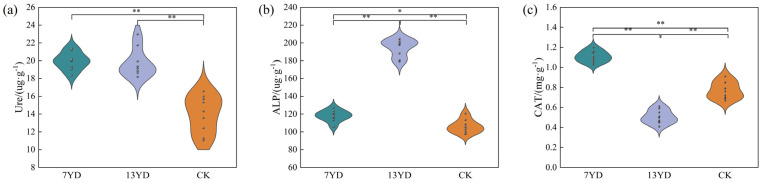
Changes in soil enzyme activities along operational duration of photovoltaic (PV) arrays (* *p* < 0.05; ** *p* < 0.01). (**a**) Ure: urease activity. (**b**) ALP: alkaline phosphatase activity. (**c**) CAT: catalase activity.

As shown in Figure 3, soil enzyme activities exhibited significant variations under PV panel shading with different operational durations in the study area. Urease activity ranged from 14.02 to 19.95 μg·g^−1^, showing a non-significant increasing trend in 7YD compared to 13YD, but was 42% higher on average in PV-shaded areas than in unshaded controls (Figure 3a). Alkaline phosphatase (ALP) activity fluctuated between 106.1 and 194.63 μg·g^−1^, demonstrating a linear increase with longer PV panel operation (*p* < 0.05; Figure 3b). Notably, catalase activity showed highly significant differences among zones (*p* < 0.01), with 7YD exhibiting a 47% increase (*p* < 0.01) compared to CK, while 13YD showed a 33% reduction (Figure 3c).

### 3.3. The Impact of Photovoltaic Panel Operational Duration on Soil Bacterial Communities and Diversity

#### 3.3.1. Analysis of Soil Bacterial Community Composition at Phylum and Genus Levels Under Photovoltaic Panels with Different Operational Durations

**Figure 4:**
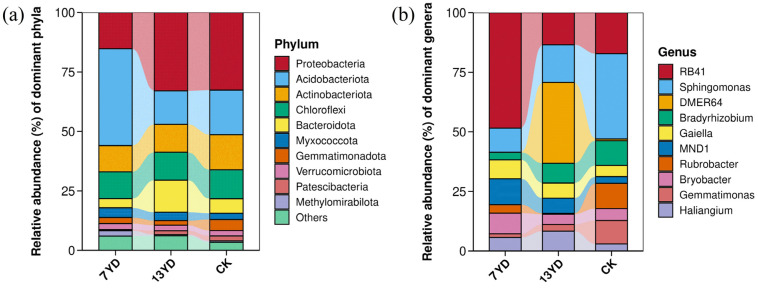
Analysis of soil bacterial community composition at phylum and genus levels. (**a**) Relative abundance at phylum level. (**b**) Relative abundance at genus level.

As shown in Figure 4, the operational duration of photovoltaic (PV) panels significantly influenced the relative abundance of soil bacterial communities at the phylum level. The top 10 dominant phyla in the study area were: *Proteobacteria* (15.2~32.9%), *Acidobacteriota* (14.1~40.7%), *Actinobacteria* (11.0~14.8%), *Chloroflexota* (11.28~12.16%), *Bacteroidota* (3.9~13.5%), *Myxococcota* (2.6~4.1%), *Gemmatimonadota* (1.9~4.6%), *Verrucomicrobiota* (2.2~2.5%), *Patescibacteria* (0.4~2.04%), and *Methylomirabilota* (0.53~3.48%). Collectively, *Proteobacteria*, *Acidobacteriota*, *Actinobacteria*, *Chloroflexota*, and *Bacteroidetes* dominated the bacterial communities (Figure 4a), accounting for over 90% of total sequences, exhibiting typical oligotrophic soil microbiome characteristics. Prolonged PV operation significantly increased the relative abundance of *Bacteroidota* but decreased *Acidobacteriota*. At the genus level, core functional taxa included *RB41*, *Sphingomonas*, *DMER64*, *Bradyrhizobium*, *Gaiella*, *MND1*, and *Rubrobacter* (Figure 4b). Notably, PV operational duration drove significant ecological niche reconstruction of key genera. The relative abundances of *RB41*, *Sphingomonas*, and *DMER64* varied markedly across PV age gradients. Extended PV operation enhanced *Sphingomonas* and *DMER64* abundances but reduced *RB41*. Compared to unshaded controls (CK), PV-shaded areas significantly decreased *Sphingomonas* abundance.

#### 3.3.2. Analysis of Soil Bacterial Alpha Diversity Under Photovoltaic Panels with Different Operational Durations

**Figure 5:**
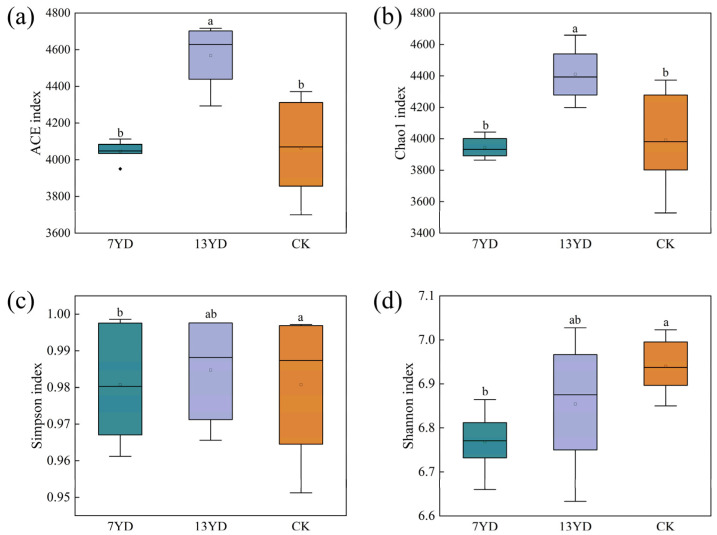
Analysis of soil bacterial alpha diversity (*p* < 0.05). (**a**) ACE index. (**b**) Chao1 index. (**c**) Simpson index. (**d**) Shannon index. ^a,b^ Different lowercase letters above the figure indicate significant differences between different treatments (*p* < 0.05).

Analysis based on alpha diversity indices revealed that photovoltaic (PV) panels of different operational durations significantly affected soil bacterial community richness and diversity by regulating microhabitat resource heterogeneity (Figure 5a–d). Both ACE and Chao1 indices increased significantly with PV panel operation time (*p* < 0.05), showing the pattern: 13YD > CK > 7YD, while Simpson and Shannon indices remained relatively stable without significant differences among durations. Compared to unshaded controls (CK), 13YD soils exhibited significant increases in ACE and Chao1 indices (*p* < 0.05), whereas 7YD soils showed non-significant decreasing trends (*p* > 0.05). Notably, 13YD soils demonstrated higher bacterial community richness and diversity than both 7YD and CK, maintaining greater species abundance.

#### 3.3.3. Analysis of Soil Bacterial OTU Composition and β-Diversity Under Photovoltaic Panels with Different Operational Durations

**Figure 6:**
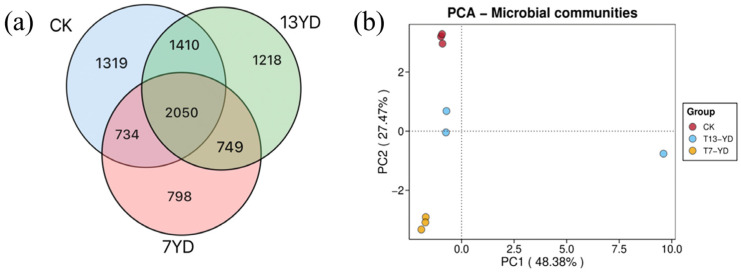
(**a**) Analysis plot of soil bacterial OTU (operational taxonomic unit) composition. (**b**) Analysis plot of soil bacterial β-diversity.

Venn network analysis (Figure 6a) revealed the OTU distribution characteristics of soil microbial communities across the study sites (7YD, 13YD, and CK), with a total of 8278 OTUs detected. Among these, 2050 core OTUs (24.8% of the total OTU library) were shared among all three sites, demonstrating the conservation of core microbiomes across different treatments. The number of shared OTUs positively correlated with community similarity. Site-specific OTUs showed gradient variations: 7YD, 13YD, and CK contained 798 (18.43%), 1218 (22.44%), and 1319 (23.93%) unique OTUs, respectively. Notably, the relative abundances of shared OTUs reached 47.33%, 37.78%, and 37.18% in 7YD, 13YD, and CK, respectively, indicating substantial similarity in soil microbial composition across the different treatment areas.

Principal component analysis (PCA) at the operational taxonomic units (OTUs) level can reveal the significant impact of the operational years of photovoltaic panels on the spatial differentiation of β-diversity in soil bacterial communities. The distance between points in the PCA plot indicates the similarity in the diversity of soil bacterial communities under photovoltaic panels of different operational ages. As shown by the principal component analysis (Figure 6b), the 7YD (7-year-old photovoltaic development) and CK (control, without photovoltaic panels) samples clustered in the third and second quadrants respectively, suggesting a relatively high similarity in their community compositions. In contrast, the 13YD (13-year-old photovoltaic development) samples were independently distributed in the fourth quadrant, indicating significant differences in soil bacterial communities between 13YD and both 7YD and CK. Additionally, the variance contribution rates of the principal coordinates along the x- and y-axes were 48.38% and 27.47% respectively, with a cumulative explanation of 75.85% of the community variation. This demonstrates a satisfactory dimensionality reduction effect of the PCA.

### 3.4. Analysis of Differences in Bacterial Community Composition Under Photovoltaic Panels with Varying Operational Years/Tenures/Service Lifespans

**Figure 7:**
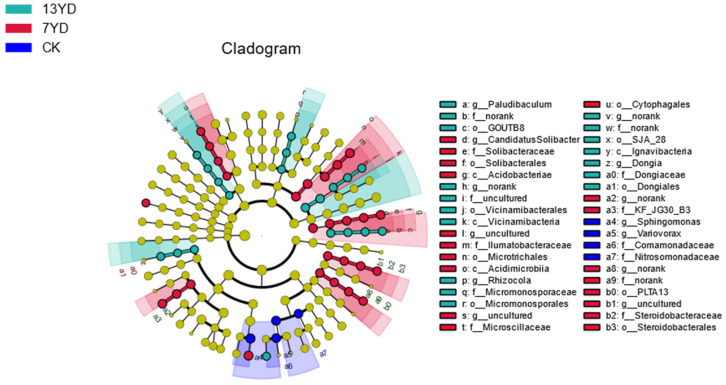
Analysis plot of differential characteristics in soil bacterial communities.

As illustrated in Figure 7, a LEfSe (linear discriminant analysis effect size) analysis based on an LDA threshold of 2 was conducted to compare groups and identify significantly different taxa contributing to the stability of soil bacterial communities across various sample plots. The results revealed the presence of distinct habitat-specific biomarkers within the soil bacterial communities of the study area, with a total of 40 significantly differentiated taxonomic units identified. Among these, seventeen taxa were significantly enriched in the 13YD (13-year-old photovoltaic development) plots, nineteen in the 7YD (7-year-old photovoltaic development) plots, and four in the CK (control, without photovoltaic panels) plots. This indicates notable disparities in bacterial community composition among the three distinct sample plot types.

Furthermore, the cladogram (hierarchical evolutionary branching diagram) provided additional insights, demonstrating that the 13YD plots were primarily characterized by the significant enrichment of *Paludibaculum*, a facultative anaerobe capable of dissimilatory iron reduction within Subgroup 3 of the *Acidobacteriaceae* family. In contrast, the 7YD plots exhibited a prominent enrichment of *Acidobacteria*, while the CK plots were dominated by the *Variovorax* genus belonging to the *Betaproteobacteria* class within the *Proteobacteria* phylum.

### 3.5. Correlation Analysis of Soil Environmental Factors, Enzyme Activities, and Bacterial Community Composition Under Photovoltaic Panels

**Figure 8:**
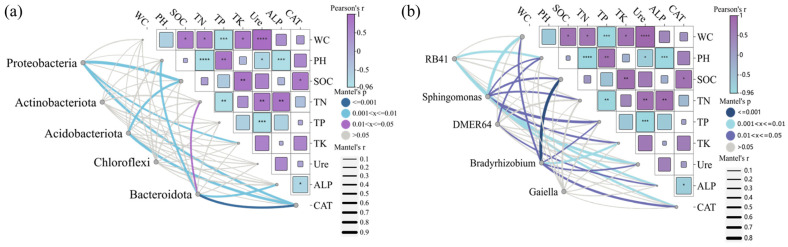
Correlation analysis plot illustrating relationships between soil environmental factors, enzyme activities, and bacterial community composition. (**a**) Phylum level. (**b**) Genus level. Asterisks indicate statistical significance levels: * *p* < 0.05, ** *p* < 0.01, *** *p* < 0.001, **** *p* < 0.0001.

The ecological interactions network tested by the Mantel test indicated that soil environmental factors and enzyme activities had significant phylum-specific driving effects on bacterial community composition. As shown in Figure 8a, at the phylum level, there were highly significant correlations (*p* < 0.01) between the abundance of *Proteobacteria* and the activities of SOC, TK, and CAT. *Acidobacteriota* abundance was likewise strongly regulated by SOC and TK, with which it was highly significantly correlated (*p* < 0.01). The main factors affecting *Bacteroidota* were PH, TN, and CAT, with highly significant correlations between *Bacteroidota* and PH and CAT (*p* < 0.01) and significant correlations with TN (*p* < 0.05). In contrast, there was no significant correlation (*p* > 0.05) between *Actinobacteria* and Chloroflexota and both soil environmental factors and enzyme activities. As shown in Figure 8b, there was a highly significant correlation (*p* < 0.01) between the abundance of *RB41* and PH and CAT at the genus level. In contrast, there were significant correlations between *Sphingomonas* and both soil environmental factors and enzyme activities, with highly significant correlations (*p* < 0.01) with WC, Ure, and ALP. In addition, *DMER64* was significantly correlated with PH and ALP (*p* < 0.05). *Bradyrhizobium* was significantly correlated with WC, Ure, and CAT, and was highly significantly correlated with SOC (*p* < 0.01), whereas there was no significant correlation (*p* > 0.05) between *Gaiella* and both soil environmental factors and enzyme activities. The global analysis showed that PH and enzyme activities were the main factors affecting the composition of bacterial communities, and their “enzyme activity–redox-nutrient coupling” mechanism provided key biomarkers for the prediction of soil microbial functions under the shade of photovoltaic panels.

## 4. Discussion

The installation and long-term operation of photovoltaic (PV) panels induce complex ecological feedback mechanisms in rocky desertification areas. We hypothesize that PV panels reduce direct solar radiation and soil temperature, thereby suppressing evaporation and enhancing rainwater accumulation in shaded zones. However, prolonged operation may limit organic matter input and weaken soil structure, gradually reducing water retention capacity over time. These microclimatic modifications trigger cascading effects on: (i) soil physicochemical properties through altered energy-water balance (Section 4.1), (ii) enzyme activities via moisture-mediated microbial regulation (Section 4.2), (iii) bacterial community composition due to niche differentiation (Section 4.3), and (iv) biodiversity patterns shaped by environmental-enzymatic coupling (Section 4.4). The following sections systematically evaluate these interactions based on 7–13 years of field evidence from karst ecosystems.

### 4.1. Effects of Photovoltaic Panel Operation Duration on Environmental Factors in Karst Rocky Desertification Regions

The installation of photovoltaic (PV) panels can alter the spatial distribution patterns of solar radiation and precipitation, leading to a restructuring of the underlying surface’s energy-water balance and subsequently driving systematic changes in soil physicochemical properties [22]. Moscatelli M C et al. conducted a seven-year field study at a coastal photovoltaic power plant in Italy, which demonstrated that PV panel installation enhanced the interaction between soil minerals and organic matter, thereby improving both soil water retention capacity and organic matter content. Furthermore, PV panel construction can alter local microclimatic conditions, primarily through modifications to evapotranspiration patterns and the redistribution of precipitation. These changes may disrupt the original hydrothermal equilibrium of the soil system, leading to significant variations in soil moisture dynamics [13]. This study found that the soil water content (WC) in shaded areas under PV panels was significantly higher than in unshaded areas, but it decreased with increasing operational of the panels. This may be attributed to the shading effect of PV panels reducing soil temperature, thereby effectively suppressing soil water evaporation. Moreover, the tilted design of PV panels facilitates rainwater accumulation in the shaded areas, further enhancing soil moisture [23]. In the short term, the combined effects of shading (reducing evaporation) and rainwater redirection can increase WC under PV panels. However, prolonged operation may limit organic matter input, reduce litterfall production due to continuous shading, and weaken soil aggregate stability, ultimately diminishing water retention capacity. As a critical physical property of soil, changes in WC influence the release of pH-related ions and the decomposition of soil nutrients [7]. In this study, the soil pH in unshaded areas was significantly higher than in shaded areas, likely because PV panels redistribute rainfall, reducing the effect on soil in shaded regions. This results in fewer OH- ions being released from limestone, leading to lower pH values under PV panels [24]. Liu Z et al. found that PV panel installation promotes organic carbon accumulation and enhances soil carbon sequestration capacity, which aligns with the findings of this study. Although PV construction may initially disturb the soil layer, the study area’s soil parent material is predominantly limestone, and the ecosystem gradually stabilizes over time [25]. The presence of PV panels may slow the decomposition and mineralization of soil organic matter, affecting physicochemical properties and nutrient cycling. Additionally, the shading effect creates cooler conditions under the panels, favoring soil carbon accumulation, thereby significantly increasing SOC content [26]. Wang F et al. reported minimal differences in TN and TK content between areas under and outside PV panels, suggesting no significant variation in soil nutrients. In contrast, this study found higher TN and TK levels in shaded areas, with TN showing an initial decline followed by an increase [2]. This could be due to the destruction of soil aggregate structure during PV construction (e.g., vegetation removal and soil compaction) [27], which may not fully recover within seven years but reaches equilibrium after 13 years of operation. Alternatively, the heterogeneous environment under PV panels may affect soil temperature, respiration, organic matter accumulation, and chemical reaction rates. Increased soil moisture also promotes the release of lattice mineral potassium, further enhancing TK accumulation [28]. These findings are consistent with previous studies, demonstrating that PV panel installation can effectively improve soil physicochemical properties and overall fertility [29]. In summary, PV panels can influence regional soil characteristics, with the extent of impact varying depending on the duration of operation.

### 4.2. Effects of Photovoltaic Panel Operation Duration on Soil Enzyme Activity in Rocky Desertification Areas

Soil enzymes, as a collective of biological catalysts, not only directly participate in key biogeochemical cycles such as organic matter decomposition and nutrient transformation, but their activity levels also serve as sensitive biological indicators of soil fertility status and ecosystem health [30]. The results of this study demonstrate significant differences in soil enzyme activity under photovoltaic (PV) panels of different operational durations. Compared to non-PV areas, PV shading enhanced soil urease (Ure) and alkaline phosphatase (ALP) activity to varying degrees, with both enzymes showing progressively higher activity as PV panel operation increased, a finding consistent with other studies [31]. This phenomenon may be attributed to the shading effect of PV panels reducing potential evaporation and altering surface microenvironments, leading to lower soil temperature and higher humidity, thereby preserving soil moisture under the panels [32]. The increased activity of urease and alkaline phosphatase could be due to elevated soil moisture promoting the growth and reproduction of microorganisms that secrete these enzymes, while also improving soil nutrient transformation efficiency [33].

On the other hand, initial soil disturbance during PV power station construction may cause a short-term decline in enzyme activity. However, with secondary vegetation recovery or adaptive microbial community succession, certain enzyme activities may gradually recover or even increase due to organic matter accumulation. Additionally, soil enzymes play a crucial role in nutrient cycling and various biochemical reactions. For example, soil catalase effectively decomposes hydrogen peroxide, preventing potential harm to soil organisms [34]. In this study, catalase activity initially increased but later decreased with prolonged PV panel operation. This trend may be linked to the gradual release of trace heavy metals from aging PV components, which can disrupt enzyme protein structures and exert toxic effects on redox enzymes like catalase, ultimately leading to reduced activity over time.

### 4.3. Effects of Photovoltaic Panel Operation Duration on Bacterial Community Composition and Diversity in Rocky Desertification Areas

As the core component of soil microbial communities, bacteria play a vital role in material cycling, energy flow, and maintaining ecological balance in terrestrial ecosystems. They drive key processes such as soil biogeochemical cycles, energy flux regulation, and ecological homeostasis maintenance [25]. The construction of photovoltaic (PV) panels significantly increased the richness and diversity of soil bacterial communities while altering their composition. *Proteobacteria*, *Acidobacteriota*, and *Actinobacteria* were identified as the dominant bacterial phyla in the soil, widely distributed across different habitats, consistent with this study’s findings, though their relative abundances showed significant variations. Due to their unique functional attributes and survival strategies, the relative abundances of dominant bacterial groups responded differently to PV shading effects over varying operational durations [35].

As metabolically versatile chemoheterotrophic bacteria, *Proteobacteria* exhibited an initial decline followed by an increase in relative abundance with prolonged PV panel operation. This trend may be attributed to the disruption of soil structure during early-stage PV construction, which temporarily compromised their habitat [36]. In this study, extended PV operation significantly increased the relative abundance of *Acidobacteriota* but reduced that of *Actinobacteria* under the panels. This divergence primarily stems from *Acidobacteriota’s* adaptability to low-temperature or acidic environments, whereas *Actinobacteria*, as representative copiotrophs, predominantly thrive in nutrient- and moisture-rich soils [37]. The study area, located in a subtropical plateau region under monsoon climate control, features abundant precipitation and strong leaching effects. In unshaded areas, precipitation-mediated neutralization lowered pH and depleted soil nutrients, creating favorable conditions for *Acidobacteriota* proliferation.

### 4.4. Effects of Environmental Factors and Enzyme Activities on Bacterial Community Composition and Diversity in Rocky Desertification Areas

Investigating the response patterns of key microbial functional guilds and their diversity to environmental factors and enzyme activities is crucial for establishing microbiological evaluation indices of soil quality and maintaining soil health [38]. Under varying conditions, the factors influencing bacterial community composition and diversity exhibit heterogeneity. In this study, the differential impacts of photovoltaic (PV) panel installation duration on soil bacterial community composition and species diversity were predominantly regulated by soil pH, total potassium (TK), and catalase (CAT) activity. As a pivotal indicator of soil chemical properties [39], soil pH significantly enhanced bacterial α-diversity and the relative abundance of copiotrophic taxa such as *Acidobacteriota* and *RB41* under PV shading, consistent with findings by Bai et al. [36], which demonstrated that PV shading modulates soil pH through altering surface hydrothermal balance, thereby generating unique microscale pH gradients that drive adaptive restructuring of soil bacterial community composition and diversity. Meanwhile, our study identified soil TK as a critical chemical factor shaping bacterial community structure and functional diversity, exhibiting a significant positive correlation with *Proteobacteria*. Potassium ions, as primary osmotic regulators in soil with high migration rates vulnerable to leaching, are strongly influenced by hydrothermal conditions, a finding corroborating the intimate relationship between soil nutrients and bacterial communities observed by Li et al. [40]. Furthermore, correlation analyses revealed significant associations between *Proteobacteria*, *Acidobacteriota*, *Actinobacteria*, and CAT activity. Philippot et al. [39] suggested that hydrothermal alterations induced by PV shading may suppress aerobic microbial respiration, thereby activating CAT activity. However, the relationship between CAT and bacterial diversity exhibits context-dependent dual effects: while short-term PV shading enhances CAT activity and promotes α-diversity, prolonged shading creates hypoxic conditions that reduce CAT enzyme activity, aligning with our study’s results. These findings underscore the necessity for future research to prioritize how PV-induced microclimatic shifts may invert the suppressive or promotive effects of soil environmental factors and enzyme activities on bacterial communities and species diversity, thereby advancing mechanistic insights into PV shading’s impacts on microclimates and soil microbiomes.

## 5. Conclusions

### 5.1. Soil Physicochemical Properties

Long-term PV operation (13 years) significantly altered soil properties, increasing water content (35%), total nitrogen (33%), and total potassium (27%), while decreasing pH (17%) and total phosphorus (21%) due to modified rainfall distribution and mineralization processes.

### 5.2. Soil Enzyme Activities

PV operation enhanced urease and alkaline phosphatase activities (42% and 47% increases, respectively), whereas catalase activity showed a time-dependent response—initially increasing by 47% in 7-year sites but declining by 33% in 13-year sites—closely linked to soil moisture and organic matter dynamics.

### 5.3. Bacterial Community Structure

PV installation induced significant bacterial community shifts, with increased *Bacteroidota* and decreased *Acidobacteriota* at the phylum level. At the genus level, *Sphingomonas* and *DMER64* abundances rose, while *RB41* declined. Long-term PV sites also hosted unique taxa, such as iron-reducing *Paludibaculum*.

Microbial α-diversity (ACE and Chao1 indices) peaked in 13-year PV areas, while β-diversity analysis revealed distinct clustering between PV and non-PV sites. Despite these changes, 24.8% of OTUs were conserved across all sites, indicating a resilient core microbiome.

### 5.4. Driving Environmental Factors

Key drivers of microbial community changes included soil pH, catalase activity, and water content. Total potassium strongly influenced *Proteobacteria* abundance, and hydrothermal balance modifications under PV panels created unique microhabitats.

In summary, this study demonstrates that PV installation establishes predictable ecological gradients in rocky desertification ecosystems, with operation duration critically shaping soil responses. These findings provide a scientific foundation for ecological risk assessment and sustainable solar energy development in fragile karst regions.

## Figures and Tables

**Table 1:** Location of study area.

Plot (Year)	PV Panel Parameters	Lat/Long	Altitude (m)	Slope Type
7 years	The front eave height of the solar panel is 1.3 m above the ground, the rear eave height is 1.9 m, and the tilt angle is 24°	103°24′41″ E, 24°50′11″ S	1892	Flat slope
13 years		103°24′27″ E, 24°50′13″ S	1892	Flat slope
CK	/	103°24′18″ E, 24°50′34″ N	1892	Flat slope

## Data Availability

The original contributions presented in this study are included in the article. Further inquiries can be directed to the corresponding author.
